# AI-enabled POCUS for breast cancer risk stratification in a resource-limited tertiary clinic

**DOI:** 10.4102/sajr.v29i1.3195

**Published:** 2025-10-09

**Authors:** Kathryn Malherbe, Francois Malherbe, Liana Roodt

**Affiliations:** 1 Department of Imaging, Faculty of Health Sciences, Malherbe Imaging Inc, Pretoria, South Africa; 2 Department of Surgery, Faculty of Health Sciences, University of Cape Town, Cape Town, South Africa; 3 Division of General Surgery, Department of Surgery, Faculty of Health Sciences, University of Cape Town, Cape Town, South Africa

**Keywords:** Breast AI, point-of-care ultrasound, breast cancer, diagnostic triage, artificial intelligence

## Abstract

**Background:**

Breast cancer remains a major public health burden in South Africa, with diagnostic delays contributing to poor outcomes. Ultrasound is effective for early detection but is limited by access and operator variability. Integrating artificial intelligence (AI) into point-of-care ultrasound (POCUS) offers a potential solution.

**Objectives:**

To evaluate the diagnostic performance of a locally developed AI-enabled POCUS system (Breast AI) in predicting malignancy among women with palpable breast abnormalities.

**Method:**

A prospective cohort study was conducted between June 2024 and November 2024 at Groote Schuur Hospital. Women aged ≥ 25 years with suspicious breast lesions underwent Breast AI ultrasound prior to biopsy. Real-time malignancy risk scores were compared with histopathological results. Diagnostic accuracy was assessed using sensitivity, specificity, positive predictive value (PPV), F1 score and area under the curve (AUC).

**Results:**

Among 159 participants, Breast AI achieved a sensitivity of 67.2%, specificity of 79.4% and PPV of 70.3% at a 51% threshold. The AUC was 0.76, reflecting moderate discriminatory performance. F1 score analysis identified 51% as the optimal cut-off (F1 = 65.7%). Benign pathologies such as fibroadenomas and fat necrosis correlated with low AI scores. A three-tiered risk model was developed: < 30% (low), 30% – 51% (intermediate) and > 51% (high risk).

**Conclusion:**

Breast AI demonstrates promising diagnostic accuracy for triaging suspicious breast lesions, particularly in resource-constrained settings.

**Contribution:**

This study provides real-world evidence supporting the integration of AI into POCUS to improve breast cancer detection and clinical decision-making in low-resource environments.

## Introduction

Breast cancer remains a major public health burden in South Africa, with women facing a 1 in 29 lifetime risk of developing the disease.^1^ Despite increasing incidence rates, delays in diagnosis and assessment, exacerbated by systemic inefficiencies and sociocultural barriers, contribute significantly to poor clinical outcomes.^2,3^

In response, South Africa’s Breast Cancer Prevention and Control Policy emphasises clinical breast examinations (CBEs) and breast self-examinations (BSEs) as essential early detection strategies, particularly in the absence of a national mammographic screening programme.^4^ While these modalities are endorsed by the National Department of Health, implementation has been hindered by limited resources, inconsistent training, and low levels of public awareness.^3^ Multimedia educational campaigns have sought to improve community engagement and encourage early presentation.^5^

Ultrasound is a critical contributory imaging modality in breast cancer detection,^6.7,8^ particularly for women with dense breast tissue or in settings where mammography access is limited. It enhances the characterisation of clinically palpable malignancies and is widely recognised as an effective adjunct to CBEs.^6,7^ Within South Africa, increasing the availability of ultrasound – especially point-of-care ultrasound (POCUS) – has been identified as a strategic priority.^8^ Conventional ultrasound typically involves a cart-based system operated in radiology suites, whereas POCUS refers to portable or handheld ultrasound devices used directly at the patient’s bedside or in primary care settings, enabling real-time clinical decision-making. However, broader integration of both modalities is constrained by disparities in equipment availability, inadequate practitioner training and workflow inefficiencies.^9,10^

The introduction of artificial intelligence (AI) to breast ultrasound holds significant promise in addressing these limitations. Artificial intelligence algorithms have demonstrated high accuracy in lesion detection, reducing observer variability and improving diagnostic efficiency.^11,12^ In South Africa, an AI-based breast ultrasound system has recently received approval from the South African Health Products Regulatory Authority (SAHPRA) as a Class B medical device. This system has been trained on more than 40 000 histologically confirmed cases and demonstrated a reported diagnostic accuracy of 97.6%.^13^ The Breast AI application, integrated with a wireless Clarius™ handheld ultrasound probe and accessible via Android, enables real-time malignancy risk prediction.^13^

This study evaluated the performance of the Breast AI system in a high-volume tertiary care setting, assessing its diagnostic accuracy and utility in predicting breast malignancies in patients presenting with palpable abnormalities. Breast AI was employed to assess malignancy risk, with predictions cross-referenced against histopathological findings. The model’s performance was evaluated using standard diagnostic metrics, namely sensitivity (the ability of Breast AI to correctly identify malignant cases), specificity (the ability of Breast AI to correctly classify benign cases) and positive predictive value (PPV) (the likelihood that a lesion flagged as high-risk by Breast AI corresponds to malignancy).

## Research methods and design

### Study design, setting and data collection

A prospective cohort study was conducted at the Groote Schuur Hospital diagnostic breast clinic from 01 June 2024 to 30 November 2024. A total of 174 participants were enrolled in this study. Fifteen patients were excluded because of the non-diagnostic nature of the biopsy specimen. Patients aged 25 years and older who presented to the breast clinic with a suspicious lesion (a palpable abnormality) requiring a biopsy, were included in the study. All biopsies were performed under ultrasound guidance. Patients excluded were those under 25 years of age, those with locally advanced tumours involving the skin, obvious inflammatory conditions (mastitis and breast abscess), and solitary axillary lymph nodes. Other exclusion criteria included patients who had a prior confirmed diagnosis of breast cancer before undergoing Breast AI analysis, had incomplete imaging data, or had no confirmed histological diagnosis after biopsy and were lost to follow-up before a final diagnosis could be determined.

The sample size was calculated using the formula for estimating a single population proportion, where *Z* is the *Z*-score corresponding to a 95% confidence level (1.96), *p* is the expected proportion of malignancy (assumed at 0.5 for maximum variability), and *d* is the margin of error (set at 0.075 or 7.5%). To account for potential exclusions and data loss, an additional 15% was added, yielding a target sample size of approximately 197. Ultimately, 174 participants were enrolled, which retained adequate statistical power for the primary diagnostic performance analyses.

All participants underwent CBE followed by imaging with the Breast AI system, which integrates real-time malignancy risk scoring using a wireless Clarius™ ultrasound probe. The AI system provided percentage-based malignancy predictions at the point of care. All imaging and diagnostic data were prospectively recorded into a secure REDCap database. Collected variables included: patient demographics (age, gender), body mass index (BMI), presenting symptoms and their duration, breast volume (estimated on ultrasound), lesion location, AI prediction percentage, biopsy method, and final histology report.

The Breast AI system uses an artificial neural network (ANN)-based deep learning model that provides real-time probabilistic classification of malignancy risk. The model was trained on over 40 000 histologically confirmed ultrasound images and is capable of feature extraction from greyscale images, enabling contextual lesion interpretation. The system differs from traditional AI tools by integrating risk prediction into a handheld point-of-care device, promoting bedside utility. Three qualified operators participated: one radiologist and two mammographers, each with 10–15 years of breast imaging experience. This POCUS-based protocol, while distinct from conventional cart-based breast ultrasound systems, ensured portable and consistent acquisition suitable for triage applications.

Ultrasound image acquisition followed standard breast scanning protocols in alignment with the American College of Radiology (ACR) practice guidelines. All examinations were conducted using a Clarius L15 HD3 handheld linear array probe (5 MHz – 15 MHz), optimised for high-resolution superficial imaging. Operators were instructed to perform systematic radial and anti-radial scans across the lesion, including full coverage in the longitudinal and transverse planes. Scan depth was adjusted to include the chest wall, and gain, and focal zones were optimised in real-time to ensure uniform echogenicity. Images were acquired with minimal probe compression to preserve lesion architecture. Each breast was scanned in a clockwise manner from the 12 o’clock position, with annotations for clock-face location, depth, size and proximity to anatomical landmarks (e.g. nipple, Cooper’s ligaments). Operators also evaluated lesion margins, echotexture and posterior acoustic features according to BI-RADS descriptors. All sonographers received standardised refresher training prior to the study to ensure consistency across image acquisition.

### Data analysis

Descriptive statistics were computed using frequencies, proportions, medians, and interquartile ranges (IQRs) where appropriate. Continuous variables were compared using Student’s *t*-test or one-way analysis of variance (ANOVA) for normally distributed data, and non-parametric equivalents (e.g. Mann–Whitney *U* or Kruskal–Wallis tests) for skewed distributions. To evaluate classification performance, confusion matrices and receiver operating characteristic (ROC) curves were constructed, and the area under the curve (AUC) was calculated. Raincloud plots were used to visualise the distribution of AI risk scores stratified by histological outcome. All statistical analyses were performed using R software version 4.3.2 (R Foundation for Statistical Computing, Vienna, Austria), and visualisations were generated using the ggplot2 and ggdist packages.

Additionally, a Breast AI-RADS (Artificial Intelligence – Reporting and Data System)–like categorisation scheme was employed to assess risk stratification thresholds. This system mirrors the BI-RADS framework, assigning Breast AI outputs, thereby standardising interpretation and supporting clinical decision-making. A confusion matrix was generated using a predefined risk threshold of 51%, identified via Youden’s Index from the ROC analysis. Lesions with AI-generated malignancy risk scores ≥ 51% were classified as ‘high-risk’, while those < 51% were deemed ‘low-risk’. This structured approach enabled a comprehensive evaluation of Breast AI’s ability to function as a diagnostic support tool, with findings compared to standard histological diagnoses. All analyses were performed using a significance threshold of *p* < 0.05.

### Ethical considerations

Ethical approval was obtained from the University of Cape Town Health Research Ethics Committee (reference number 371/2024), and informed consent was acquired from all participants in advance. All data were entered into a password-protected REDCap database and exported anonymously to an Excel spreadsheet for analysis.

## Results

A total of 174 participants were enrolled in this study. Fifteen patients were excluded because of the non-diagnostic nature of the biopsy specimen. The median age of the cohort was 48 years (IQR 38–60), with a median BMI of 29.5 kg/m^2^ (IQR 25–34), placing the group in the overweight category. The median breast volume was 494 cc (IQR 325.5–699.5).

With the risk prediction threshold suggested at 51% for the Breast AI system, it correctly identified 45 malignant cases (true positives) and 73 benign cases (true negatives). It also incorrectly flagged 19 benign lesions as high-risk (false positives) and failed to detect 22 malignant cases (false negatives). These values were used to derive the system’s core diagnostic performance metrics. The Breast AI system demonstrated moderate performance when benchmarked against clinical standards (Table 1).

**TABLE 1:** Comparative diagnostic performance metrics.

Metric	Breast AI (%)	Diagnostic benchmark (%)
Sensitivity	67.2	85.0
Specificity	79.4	80.0
Positive predictive value	70.1	70.0

AI, artificial intelligence.

The F1 score serves as a balanced measure of diagnostic performance, particularly when both false positives and false negatives carry clinical consequences. It is defined in Equation 1 as:


$$\begin{array}{l} F_{1}=2\times (Precision\times Recall)/(Precision+Recall)\\ F_{1}=2\times (70.3\times 67.2)\;4724,16/(67.6+67.2)\approx 70.1\%\\ Precision=TP/(TP+FP)=45/(45+19)\approx 70.3\%\\ Recall=TP/(TP+FN)=50/(50+21)\approx 70.4\% \end{array}$$ [Eqn 1]

Further analysis of diagnostic performance by lesion laterality revealed asymmetry in system performance, with AI sensitivity and specificity higher for right-sided lesions. Table 2 highlights the performance discrepancies between left and right breast lesion detection.

**TABLE 2:** Diagnostic performance by lesion laterality.

Side	True positives	False negatives	True negatives	False positives	Sensitivity (%)	Specificity (%)
Right	12	2	15	2	85.7	88.2
Left	10	7	17	3	58.8	85.0

**FIGURE 1:**
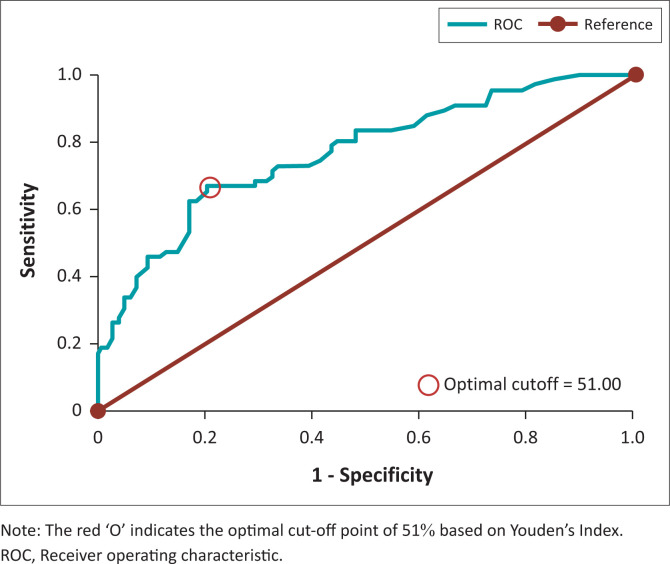
Receiver operating characteristic (ROC) curve for breast artificial intelligence system: ROC curve (area under the curve: 0.768).

The ROC curve for the Breast AI system yielded an AUC of 0.76, indicating moderate to strong discriminatory power between benign and malignant lesions (Figure 1). The optimal malignancy risk threshold of 51%, determined by Youden’s Index, was used for binary classification throughout this study (Figure 1).

**FIGURE 2:**
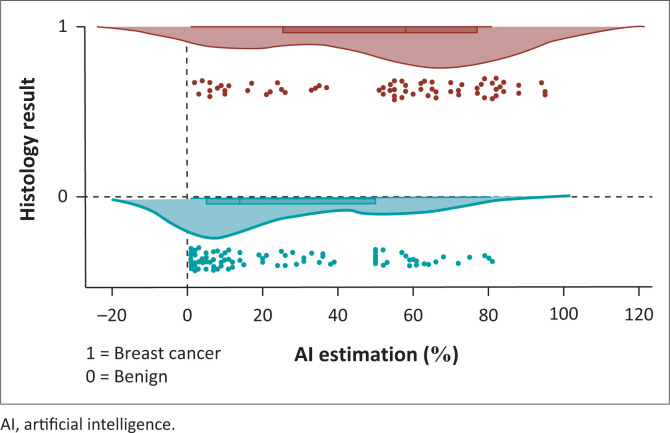
Raincloud plot interpretation of artificial intelligence estimation (%) by histology result.

The distribution of AI-generated malignancy risk scores is illustrated in the raincloud plot (Figure 2). The results demonstrated a bimodal distribution, with most benign lesions clustering below 30% and malignant lesions predominantly distributed above 51%. A diagnostic ‘grey zone’ was identified between 30% and 51%, where score overlap created potential classification ambiguity.

**FIGURE 3:**
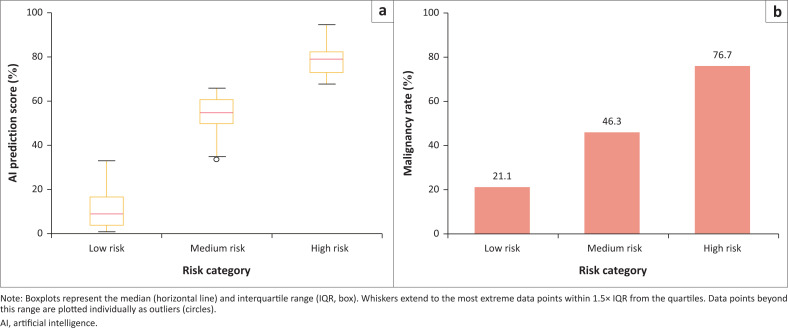
(a) Artificial intelligence (AI) prediction score versus (b) histology-confirmed malignancy rates by risk stratification.

**FIGURE 4:**
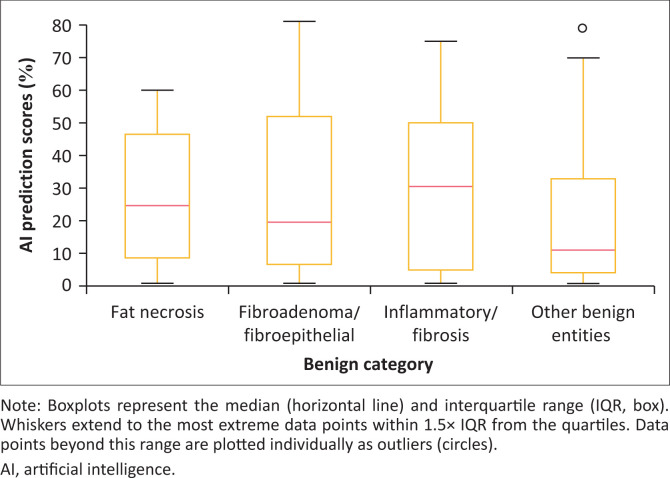
Artificial intelligence risk prediction score index for a benign diagnosis.

This suggests the potential clinical benefit of a three-tiered risk stratification system, categorising cases into low-risk (< 30%), intermediate-risk (30% – 50%) and high-risk (≥ 51%) categories to support more nuanced triage decisions. This is seen in Figure 3:

The benign lesions were classified into four groups based on final histology: (1) abscesses or inflammatory conditions and fibrosis, (2) fibroadenomas or other fibroepithelial lesions, (3) fat necrosis and (4) all remaining benign entities. ANOVA revealed no statistically significant differences among the groups (*p* = 0.79). Corresponding box plots are presented in Figure 4. Further breakdown of the benign entities termed as other is seen in Table 3.

**TABLE 3:** Confidence interval rate for benign findings versus risk prediction score.

Benign category	Count	Mean	Standard	s.e.	95% CI	
					lower	upper
Fat necrosis	10	27.1	21.5	6.8	13.8	40.4
Fibroadenoma/fibroepithelial	38	27.8	25.7	4.2	19.6	36.0
Inflammatory/fibrosis	22	29.7	25.7	5.5	18.9	40.4
Other benign entities	37	22.3	23.4	3.9	14.7	29.9

CI, confidence interval; s.e., standard error.

To evaluate variation in AI-predicted malignancy scores among revised benign histology categories, a one-way ANOVA was conducted. The analysis revealed no statistically significant differences between groups (*F* = 0.52, *p* = 0.67), suggesting that the AI system assigned comparable malignancy scores across distinct benign pathologies.

## Discussion

This study demonstrates that the Breast AI system provides clinically meaningful support in identifying high-risk breast lesions, achieving a sensitivity of 67.2%, specificity of 79.4% and a PPV of 70.3% at a 51% malignancy threshold. These results underscore the system’s potential utility as an adjunctive triage tool, particularly within resource-limited or high-volume clinical environments, by accurately flagging the majority of histologically confirmed malignant lesions for expedited referral.

Lateral discrepancy, as indicated in Table 2, warranted inclusion in this study as it may reflect real-world scanning challenges or biases introduced during model training. Prior research has shown anatomical and technical differences in image acquisition between breast sides – often influenced by operator handedness and transducer angulation – which can affect lesion visibility and diagnostic accuracy.^14,15^ Moreover, AI models may be impacted by such confounding variables if laterality distribution is unbalanced in training data, potentially leading to skewed predictive performance.^16^ Reporting side-specific outcomes, therefore, offers valuable insight into generalisability and guides future refinements in AI training protocols to ensure equitable diagnostic performance.

The system’s discriminatory capacity was further supported by the ROC curve analysis, which yielded an AUC of 0.76, indicating acceptable diagnostic performance. Using Youden’s Index, the optimal malignancy risk threshold was initially identified at 51%, providing a pragmatic balance between sensitivity and specificity. Further recalibration using F1 score maximisation reaffirmed 51% as the optimal threshold, at which the model achieved its maximum F1 score of 65.7%. This suggests that a slightly lower threshold may better balance false positives and false negatives, depending on clinical context or risk tolerance. Area under the curve values in the range of 0.70–0.80 are generally considered clinically acceptable and support the feasibility of AI integration into diagnostic triage workflows, especially in settings with limited radiologist availability. A large, international study evaluating an AI system for breast cancer screening similarly concluded that AUCs within this range can justify clinical utility, particularly for triage or augmentation roles.^17^

**FIGURE 5:**
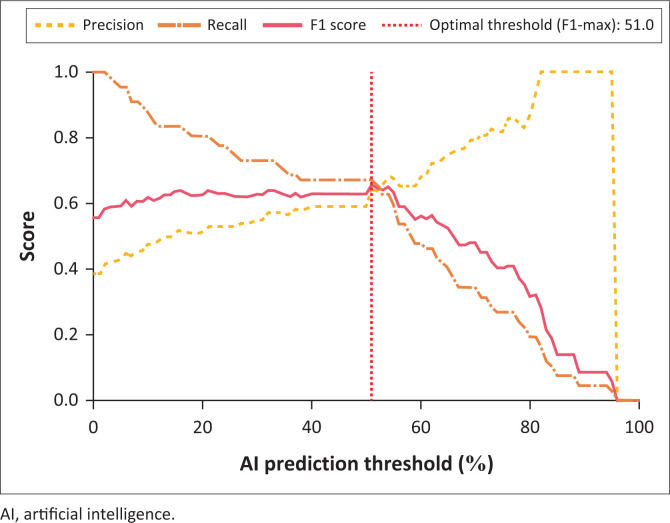
Precision-recall and F1 score threshold assessment.

A proposed three-tier risk stratification model – low-risk (≤ 30%), intermediate-risk (31% – 50%) and high-risk (≥ 51%) – remains clinically relevant. The analysis confirmed that benign lesions predominantly clustered in the low-risk range, while most confirmed malignancies scored above the 51% threshold (Figure 5). However, the intermediate ‘grey zone’ (31% – 50%) remains diagnostically ambiguous and may benefit from closer clinical correlation and short-interval follow-up rather than immediate intervention.

To further optimise the diagnostic balance between sensitivity and specificity, a performance curve was generated plotting F1 score, precision, and recall across AI-predicted malignancy thresholds (Figure 5). This analysis identified an optimal threshold of 51%, where the model achieved its maximum F1 score of 65.7%. The F1 score integrates both precision and recall, making it particularly relevant in oncologic triage where false negatives and false positives carry clinical consequences. This approach aligns with findings from Rodríguez-Ruiz et al.,^18^ who reported that F1-maximised thresholds yielded improved triage performance over fixed cut-offs in mammographic AI systems. Similarly, previous studies^16,19^ have emphasised the importance of threshold tuning in resource-constrained settings, where overly conservative thresholds may overburden referral systems, while overly liberal thresholds may compromise early detection. Compared to prior AI ultrasound studies reporting F1 scores between 68% and 75% in curated cohorts,^16,19^ the Breast AI system’s current performance (F1 = 65.7%) reflects a reasonable balance under real-world conditions, particularly given the diverse case mix and imaging variability in this dataset. Continued refinement of threshold strategies – potentially incorporating cost-sensitive or patient-prioritised decision rules – may further enhance clinical integration.

While overall performance metrics are promising, sensitivity remains lower than the 85% – 90% benchmarks commonly reported in curated datasets for AI-based ultrasound tools. Prior studies have documented higher sensitivity (76.9% – 85.7%) and specificity (89.2% – 96.1%) values in more controlled environments.^17,20,21^ This performance gap may reflect real-world variability in image quality, operator technique and case complexity, highlighting the need for ongoing algorithmic refinement and robust multicentre validation.^22,23^

Importantly, the Breast AI system exhibited a relatively consistent pattern in its assessment of benign lesions. Subgroup analysis of common benign histologies – including fibroadenomas, fibroadipose tissue and benign breast parenchyma – showed mean AI-predicted malignancy scores ranging from 16.7% to 23.8%, with relatively narrow confidence intervals (e.g. fibroadenoma/fibroepithelial: 23.8%, 95% CI: 16.2% – 31.4%). Slightly higher mean scores were noted for fat necrosis (27.1%, 95% CI: 17.8% – 36.4%) and fibrocystic/inflammatory lesions (30.6%, 95% CI: 21.7% – 39.4%), potentially because of sonographic overlap with malignant features. However, a one-way ANOVA showed no statistically significant differences among these groups (*F* = 0.52, *p* = 0.67), suggesting that the AI does not systematically overestimate malignancy risk within the benign spectrum. While this result should not be interpreted as evidence of equivalence, it offers preliminary support for the system’s consistency across benign subtypes. Further multicentre studies are warranted to evaluate reproducibility and generalisability in varied imaging environments.^24,25,26,27^

A focused review of false positives further supports this interpretation. Lesions such as fibroadenomas, chronic abscesses and postsurgical fibrosis were frequently flagged as high-risk because of complex echogenic patterns mimicking malignancy. These misclassifications likely reflect a conservative, safety-oriented bias rather than model error, consistent with findings from other AI-based diagnostic systems.^18^ In oncologic triage, such cautious classification may be clinically acceptable, provided it is balanced by a low rate of missed malignancies.^28,29,30^

To enhance Breast AI’s diagnostic performance and clinical utility, several refinements are recommended. Incorporating a dynamic threshold model guided by F1 maximisation or cost-sensitive adjustments could improve triage accuracy. Standardising image acquisition protocols and implementing quality control measures may help mitigate inter-operator variability. Additionally, integrating supplemental data such as Doppler flow, elastography, and relevant clinical metadata (e.g. hormonal status, genomic markers) may improve context-aware decision-making. Finally, prospective multicentre validation will be essential for confirming system generalisability and supporting regulatory approval.^31,32^

**FIGURE 6:**
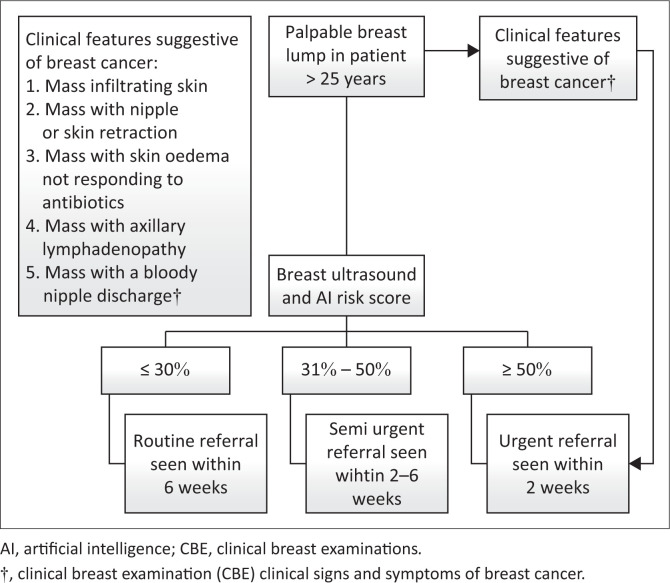
Risk-stratified clinical triage model for breast artificial intelligence.

Based on the diagnostic performance of the Breast AI system and threshold optimisation findings, we propose a three-tiered triage model to streamline clinical workflow and resource allocation, as illustrated in Figure 6:

1. *Low-risk lesions (< 30%)* may be referred for routine follow-up or annual CBE, thereby reducing unnecessary imaging or biopsy.
2. *Intermediate-risk lesions (30% – 50%)* represent a diagnostic grey zone and may benefit from short-term re-evaluation, adjunctive imaging, or clinical reassessment within 6–12 weeks.
3. *High-risk lesions (≥ 51%)*, as supported by F1 score optimisation and malignancy clustering, should be prioritised for urgent referral to diagnostic centres.

This stratified triage pathway offers a pragmatic framework for efficient resource use while maintaining diagnostic vigilance. When deployed at the point of care, Breast AI may support timely clinical decision-making and help reduce delays in cancer diagnosis, particularly in underserved or high-throughput healthcare settings.^33^

## Conclusion

The Breast AI system demonstrates promising diagnostic performance, achieving a sensitivity of 67.2%, specificity of 79.4%, PPV of 70.3%, and an F1 score of 65.7% under real-world clinical conditions. These results highlight the system’s potential utility as a triage and risk stratification tool in breast cancer care, particularly in settings where radiologist availability is limited and diagnostic backlogs are common. Its ability to identify higher-risk lesions and prioritise them for further investigation may help streamline referral workflows and reduce diagnostic delays.

Nonetheless, these findings must be interpreted within the context of the study’s limitations, including a modest sample size, imaging heterogeneity and the absence of external or temporal validation. While subgroup analyses suggest consistency in benign lesion scoring and support a three-tier threshold-based triage approach, diagnostic ambiguity within the intermediate-risk range underscores the need for continued clinical oversight.

To enable broader clinical adoption, the Breast AI system will require further algorithmic refinement, standardisation of imaging protocols, and prospective validation across diverse clinical settings. These efforts are critical to ensuring reproducibility, regulatory compliance and clinical trustworthiness. If effectively integrated into routine workflows, Breast AI has the potential to enhance early detection and reduce delays in diagnosis – especially in underserved or high-throughput healthcare environments.
